# Tree ring width and isotope ratios show that high temperatures exceed pollution effects on urban trees: evidence in *Pinus pinea* in Firenze and Pisa, Central Italy

**DOI:** 10.1007/s11356-025-37218-1

**Published:** 2025-11-21

**Authors:** Lucia Mondanelli, Paolo Cherubini, Fabio Salbitano, Matthias Saurer, Lukas Wacker, Claudia Cocozza

**Affiliations:** 1 Department of Agriculture, Food, Environment and Forestry (DAGRI), University of Firenze, Via San Bonaventura 13, 50145 Florence, Italy; 2 Swiss Federal Research Institute WSL, Zürcherstrasse 111, 8903 Birmensdorf, Switzerland; 3 Department of Forest and Conservation Sciences, The University of British Columbia, 2424 Main Mall, Vancouver, BC V6T 1Z4 Canada; 4 Department of Agricultural Science, University of Sassari, Viale Italia 39/a, 07100 Sassari, Italy; 5 Ion Beam Physics, Eidgenössische Technische Hochschule Zürich, Otto-Stern-Weg 5, 8093 Zurich, Switzerland

**Keywords:** High temperatures, Low precipitation, Traffic emissions, Dendroecology, Stable isotopes, Radiocarbon, Urban forestry

## Abstract

Urban areas typically experience higher temperatures and reduced precipitation compared to periurban environments, conditions that may strongly influence tree performance. To assess these effects, we studied *Pinus pinea* in Firenze and Pisa, integrating dendroecological and stable isotope analyses. Ring-width index data did not reveal significant differences in radial growth between urban and periurban trees, suggesting that growth responses alone may underestimate urban stress. In contrast, isotopic analyses provided clearer evidence: δ^13^C and δ^18^O values indicated marked water stress in urban trees, particularly in Firenze, while δ^15^N and F^14^C suggested minimal incorporation of traffic-derived nitrogen or fossil CO_2_, likely due to the protective role of urban parks. These findings highlight an apparent discrepancy, where tree growth suggests resilience, while physiological signals reveal stress linked to urban microclimates. This emphasizes the value of combining multiple indicators to capture tree responses in complex environments. Overall, our results suggest that urban climate exerts a stronger influence on *P. pinea* than localized traffic emissions, with implications for tree vitality and ecosystem services. Species selection for urban forestry should therefore account for microclimatic constraints to ensure long-term tree performance under ongoing urbanization and climate change.

## Introduction

Urban trees have received increasing attention in recent years due to their ability to enhance urban biodiversity, mitigate the urban climate, e.g., the heat island effect, reduce air pollution, and provide ecosystem services (Kowarik 2011). However, the significance of urban trees’ impact on the urban environment is directly linked to their physiological conditions, growth, and vitality (Czaja et al. 2020; Sjöman et al. 2015). Although numerous studies have demonstrated the positive impacts of trees on the urban climate and environment, less is understood about the influence of adverse site conditions on tree growth and health (Moser-Reischl et al. 2019). Many aspects of their functioning in urban environments remain almost unexplored (David et al. 2018). In fact, it has been a long-standing and widely accepted belief that trees in urban environments grow more slowly than those in rural areas due to the increased environmental stresses, such as higher temperatures, lower air humidity, and reduced soil moisture (Quigley 2002). In addition, Warner et al. (2024) found that heat and drought stress significantly influence tree growth at higher latitudes and cooler climatic conditions, highlighting the higher sensitivity of urban trees to climate warming in various cities across the United States. However, recent studies challenge this assumption (Smith et al. 2019; Sonti et al. 2019). A study conducted in New York City demonstrated that tree seedlings grown in urban areas exhibited growth rates twice as fast as those in rural areas (Gregg et al. 2003). Similarly, Briber et al. (2015) reported that urbanization in Boston largely accelerated tree growth in remnant forests. In the end, Imhoff et al. (2004) examined the vegetation index—an integrative measure of vegetation productivity—across urban, suburban, and rural settings, concluding that urbanization can either enhance or inhibit vegetation growth depending on the specific location and the city’s climatic background.

Trees living in urban areas face more critical environmental challenges than trees living in forests (Czaja et al. 2020), including soil sealing resulting in lower gas diffusivity and respiration rates (Fini et al. 2022; Weltecke & Gaertig 2012), limited access to water and nutrients (Yu et al. 2018), higher temperatures and the escalating urban heat island effect (Sonti et al. 2019; Sensuła and Wilczyński 2022), and increased exposure to traffic and pollution, like NO_X_ (Ferrini et al. 2014; Sensuła et al. 2015). In addition, climate change will significantly impact cities with a Mediterranean climate (Ali & Cramer 2022). Higher temperatures and lower precipitation will exacerbate the urban heat island (UHI) effect in these cities compared to surrounding areas. Understanding how the urban climate and environment affect tree growth and physiology is crucial for future adaptive urban planning and management, as well as for planning tree planting and ensuring healthy trees that provide ecosystem services (Moser et al. 2017).

In this context, tree rings, formed annually by trees in temperate climate regions, serve as valuable tools for reconstructing historical changes in the atmospheric environment (Cernusak and English 2015; Babst et al. 2018; Pearl et al. 2020). Their chemical and physical characteristics are widely used as indicators of the environmental conditions in which trees have grown, contributing significantly to discussions about the influence of environmental changes on tree growth and vitality (Cherubini et al. 2021). In the Mediterranean region, studies have shown that a positive moisture balance, low summer temperatures, and winter–spring rainfall enhance radial growth—particularly in species such as *Pinus pinea L*. in Italy—while high spring temperatures have a negative effect (Piraino et al. 2013; Mechergui et al. 2021).

In addition to assessing tree growth patterns, tree-ring stable isotopes are often used to retrospectively evaluate the impact of environmental disturbances on tree vitality (McCarroll and Loader 2004; Babst et al. 2018; Shestakova and Martínez-Sancho 2021) and offer complementary information to the tree-ring width (Siegwolf et al. 2022). Trees serve as reliable indicators of environmental health by storing information about their growth environment. Under stress conditions such as drought, plants modify carbon isotope fractionations during photosynthesis, resulting in relatively higher δ^13^C values in leaves (Farquhar et al. 1989) and tree rings (Leavitt 2013). Similarly, δ^18^O primarily reflects the oxygen isotope ratio of precipitation or of the main water source, and is associated with temperature, humidity, atmospheric circulation patterns, and leaf evaporation processes (Siegwolf et al. 2022). Nitrogen isotopes are more closely related to the nutrient cycle and nitrogen pollution, with positive δ^15^N values (up to + 7.7‰) indicating constrained environments due to anthropogenic factors such as vehicle emissions (Siegwolf et al. 2022). Tree-ring data may capture variations in the nitrogen cycle or reflect successive patterns in nitrogen cycling, but the underlying causes of these changes are highly complex (e.g., pollution, soil microbial processes). Accurate interpretations demand a thorough understanding of the specific environmental conditions in which the trees are growing. Due to the reduced isotopic signals resulting from nitrogen remobilization within the tree and the complex enzymatic fractionations occurring during nitrogen assimilation, attempts to quantify human impacts on the forest nitrogen cycle through tree rings or leaves may be limited. However, on a more optimistic note, it seems feasible to identify and date disturbances in the forest nitrogen cycle by analyzing δ^15^N patterns in tree rings (Siegwolf et al. 2022). Moreover, in evaluating δ^15^N values, a mixing model with two components was assumed—background air and polluted air—which affect the tree-ring values (Saurer et al. 2004). Recent studies have shown an enrichment in tree-ring δ^15^N values due to urbanization, while nitrogen fertilization near motorways inhibits growth (Siegwolf et al. 2022). Additionally, radiocarbon (F^14^C) analysis of tree rings can provide insight into the quality of the air taken up by trees, and the influence of fossil fuel sources of CO_2_ in the urban atmosphere, as it is depleted in fossil fuels but normally present in natural atmospheric carbon dioxide (Alessio et al. 2002; Rakowski et al. 2008, 2010; Beramendi-Orosco et al. 2018; Sensuła et al. 2021; Kontuľ et al. 2022).

The study aimed to assess the effects of the urban environment, considering weather conditions and traffic exposure, on *Pinus pinea* trees in two cities of Central Italy, Florence and Pisa, comparing urban and peri-urban sites. We investigated whether, in a Mediterranean environment, urban trees show lower or higher tree growth compared to peri-urban trees, and whether the highest maximum temperature peaks and lowest precipitation levels—relative to surrounding areas—affect tree growth and physiology. To address this, we analyzed ring width as well as tree-ring δ^13^C and δ^18^O isotope ratios. Moreover, assuming higher air pollution levels in urban areas due to vehicular traffic compared to peri-urban areas, we aimed to assess whether and how urban trees have recorded any signal of this, using tree-ring δ^15^N and F^14^C isotopes, which are widely used in the literature as proxies for investigating pollution levels (Saurer et al. 2004; Savard et al 2009; Doucet et al. 2012; Djuricin et al. 2012; Niu et al. 2024). The results contribute to a better understanding of tree health, a crucial challenge in monitoring the context of “re-greening the cities,” and to promote trees as valuable tools for assessing and guaranteeing air quality in urban areas.

## Materials and methods

### Study areas and selected species

The study areas are parks located close to and within two cities in Tuscany, Italy: Firenze and Pisa, with populations of 365,756 and 89,565 inhabitants, respectively (www.istat.it, 2025). The sites are located in central Italy on the Tyrrhenian side, characterized by relatively mild winters and hot, sunny summers. The average temperature of the coldest month (January) is 6.5 °C, while that of the hottest month (August) is 25.5 °C. The averages were calculated for the period from 1991 to 2023, using the available data for the Tuscany region. The precipitation pattern slightly deviates from the typical Mediterranean climate, as the summer minimum is present but less pronounced. Additionally, rainfall increases slightly in spring compared to winter. Autumn is the wettest season, with an annual total of about 870 mm of rainfall.

Following the United Nations’ Food Agriculture Organisation (F.A.O.) definition of urban and periurban forests, an urban area and a periurban area were selected in each city. At each site, the selected species was *Pinus pinea* L., an iconic Mediterranean species native or naturalized in southern Europe (Mechergui et al. 2021).

The periurban parks, Poggio Valicaia in Firenze (“Fi-PU,” located at 43.703 N, 11.145 E) and San Rossore in Pisa (“Pi-PU,” located at 43.721 N, 10.293 E), consist of public parks that contain *P. pinea* stands once managed for the production of edible pine nuts. Nowadays, in Fi-PU and Pi-PU pine is about 100 years old, with some broadleaf trees, especially oaks (*Quercus* ssp.) present at Fi-PU. The two urban sites, “Fi-UR” in Firenze (43.782 N, 11.216 E) and “Pi-UR” in Pisa (43.704 N, 10.425 E), are prominent urban parks along the Arno River where *Pinus pinea* is abundant. In Fi-UR, *P. pinea* is found as single trees and in pure groups spread throughout the area. In Pi-UR, *P. pinea* is arranged in multiple lines, forming a pure stand. These sites play essential ecological, landscape, and recreational roles, serving as invaluable assets for the residents of each city.

At each of the four sites, 15 trees were selected based on their apparent healthy condition: individuals showing no visible signs of disease or injuries on the trunk or in the crown, with straight trunks and well-balanced crowns were selected. The selected trees at all four sites belonged to the dominant canopy layer, with the following mean diameters: at Fi-PU 31.20 cm (± 5.3), Fi-UR 38.74 cm (± 8.9), Pi-PU 49.98 cm (± 4.72), and Pi-UR 53.54 cm (± 7.54), showing comparable dimensions between urban and peri-urban areas in both cities. Furthermore, in all cases, only trees within the stand were sampled, avoiding edge trees.

### Meteorological data and climate analysis

A climatic assessment of the study sites was performed for the period 1901–2022. Due to the absence of continuous climate data from stations near the studied sites, meteorological gridded data from the Climate Downscaling Tool (ClimateDT) were used. This system operates on a 1-km grid using a combination of CRU-TS (Climatic Research Unit gridded Time Series) for historical climate data (1901 to present) (https://www.ibbr.cnr.it/climate-dt/). Meteorological data included three monthly variables: minimum and maximum temperature, and total precipitation. Climate data are also affected by long-term trends. In this study, only high-frequency changes were of importance, and we detrended the climate variables using a 30-year smoothing spline with a 50% frequency cutoff in R using the dpIR package.

### Dendrochronological analysis

In January 2023, two increment cores, 5 mm in diameter, were collected at breast height (1.30 m) from 15 trees per site. Core surfaces were prepared using a sledge microtome (Gärtner & Nievergelt 2010), and images were captured with a digital camera (Canon EOS 5DSR) and a 100 mm macro lens (https://www.wsl.ch/en/services-produkte/skippy/) at a resolution of 5950 dpi. Ring widths were measured using CooRecorder v8.9 (Cybis Electronics, Sweden), and the tree-ring width series were validated using statistical methods in TSAP-Win software (Frank Rinn, Heidelberg, Germany). In particular, we used the Gleichläufigkeit values and the Student’s *t*-test at a significance level of *p* < 0.05 and *n* = 30 for each site. The ring-width chronologies were transformed into ring-width indices (RWI), and detrending was performed using a 30-year smoothing spline with a 50% frequency cutoff in R using the dpIR package.

### Sample preparation and stable isotopic measurements

For each site, five cores were randomly selected for isotopic analyses. They covered the period of 70 years (from 1953 to 2022) and exhibited an acceptable GLK value (> 60%) obtained by cross-dating. The 70-year period was selected by considering the oldest trees in the two urban sites.

For each core, 14 samples were analyzed, each consisting of a pool of five consecutive rings, e.g., 1953–1957, 1957–1962, and so on, up to the most recent period, 2018–2022. It was found that developing records by analyzing δ^13^C and δ^18^O in successive 5-year groups (pentads) from individual trees could produce a reliable “smoothed” isotopic chronology (Boettger and Friedrich 2009; Leavitt 2010)**.** Wood cores were cleaned of N-mobile compounds using Soxhlet equipment with organic solvents like alcohol (Saurer et al. 2004). Samples of five consecutive annual rings were prepared for all 20 cores. The wood samples were milled using a centrifugal mill and weighed in silver capsules (1 mg for δ^13^C and δ^18^O, and 15 mg for δ^15^N). δ^13^C and δ^18^O analyses were performed after thermal conversion to CO at 14.20 °C on a deltaXP isotope-ratio mass-spectrometer (Thermo, Bremen, Germany). While the traditional method for the analysis of δ^13^C is via combustion resulting in CO_2_, it has been shown that a reliable δ^13^C analysis is possible from CO after pyrolysis considering appropriate corrections and calibrations (Saurer et al. 2024). We applied a two-point calibration for each run based on materials with known δ^13^C-values, which resulted in a precision of 0.2‰ for δ^3^C and 0.3‰ for δ^18^O (standard deviation of repeated analyses). Standards used were IAEA (International Atomic Energy Agency Standards) benzoic acids IAEA-601 (δ^13^C =  − 28.81‰, δ^18^O = 23.14‰), IAEA-602 (δ^13^C =  − 28.85‰, δ^18^O = 71.28‰), sucrose IAEA-C-6 (δ^13^C =  − 10.80‰, δ^18^O = 32.14‰) and internally produced wood materials as quality control. δ^15^N values were determined after combustion under excess oxygen at 1020 °C and reduction with copper at 600 °C to N_2_ followed by isotope analysis (HS2022, Sercon, Crewe, UK), with a precision of 0.3‰ based on the standard deviation of repeated analyses. This analysis also provided the nitrogen content of the samples in %. Standard materials used were caffeine IAEA-600 (δ^15^N = 1.0‰), ammonium sulfate IAEA-N-2 (δ^15^N =  + 20.41‰), l-glutamic acid IAEA δ^15^N = 47.57‰), and internally produced reference materials from wood as quality control.

Tree-ring δ^13^C values for each sample and core were corrected for the Suess effect (Belmecheri & Lavergne 2020). In evaluating δ^15^N values, when the model proposed by Saurer et al. (2004) that considers the two components, background air and polluted air, holds true, a negative relationship between δ^15^N of the tree rings and the inverse of the N-concentration (1/N) should be observed, as expressed by the general form: δ^15^N =  − *a* (1/N) + *b*. Extrapolating the N concentration to high values (1/N ~ 0) yields δ^15^N = *b*, which is the isotope ratio of the pollution.

### Radiocarbon measurements

In our study, we considered three trees per site and focused our analysis on the outermost and innermost growth rings, spanning from 2018 to 2022 and from 1952 to 1957, respectively. To extract the holocellulose from the wood samples, we utilized the BABAB protocol (Wacker et al. 2010), which involves a sequential base-acid–base-acid bleaching process. Between each step, the samples underwent thorough rinsing with de-ionized water to maintain near-neutral pH conditions.

For the graphitization process, we employed the automated graphitization equipment “AGE”(Wacker et al. 2010). Wood samples were weighed into tin capsules and combusted in an elemental analyser to produce carbon dioxide, which was then transferred to the graphitization reactor. The CO_2_ was trapped on zeolite while the carrier gas (helium) was removed. The CO_2_ was reduced with H_2_ on an iron catalyst. The formed graphite was finally pressed into target holders for ^14^C measurement using a small 200 kV accelerator mass spectrometry (AMS) device MICADAS (Synal et al. 2007), which detects specific elements based on their atomic weights after destroying any molecular interference in the gas ionization detector, where single ions are counted. For radiocarbon (^14^C) analysis using the MICADAS system, samples were placed in cassettes, each holding 22 positions. Alongside the wood samples, each cassette included three processing blanks, two processed secondary pine standards, and five OX-II standards, 1.3407 ± 0.0005 F14C (δ^13^C, − 25.0‰), for normalization. High-precision measurements at ETH Zurich take approximately 1 h per sample, divided into ten intervals of 50 s each.

The OX-II standards typically produce around 500,000 counts (with C^−^ ion currents of 50 µA), while the samples generate approximately 300,000 counts, resulting in a counting statistical uncertainty of less than 2‰. Data processing and evaluation are conducted using the BATS software (Wacker et al. 2010), which also enables statistical verification of the measured data. To correct for background levels, the ^14^C counts were adjusted using the processing blank (F^14^C, 0.0035 ± 0.004, *n* = 6) and normalized with OX-II (*n* = 5) standards. Additionally, an extra uncertainty of 1‰ was included, based on long-term laboratory data from measurements on processed secondary wood standards. The radiocarbon values are presented in F^14^C, not subjected to misinterpretations in the literature, and are recommended for use with post-bomb samples (Stenström et al. 2011).

### Statistical analysis

The relationship between tree-ring width and monthly and seasonal climate was investigated using Pearson’s correlation, with analyses performed using ggplot2 (Wickham 2016), dpIR (Bunn 2010), and treeclim (Zang and Biondi 2015) packages in R version 4.2.3. The Shapiro–Wilk test indicated that the data did not follow a normal distribution. Therefore, the nonparametric Mann–Whitney test was used to assess differences in δ^13^C, δ^18^O and δ^15^N values between the urban and periurban sites in each city. Pearson’s correlation was also applied to examine the relationships between all isotopes and RWI and climate data, as well as to analyze the association between δ^15^N and 1/N-concentration (%). The differences in F^14^C between urban and peri-urban sites across two distinct periods were examined using ANOVA, followed by Tukey’s test as a post hoc analysis. The latter analysis was conducted using the OriginPro8 program (OriginLab Corporation, Northampton, UK).

## Results

### Climate data

**Fig. 1:**
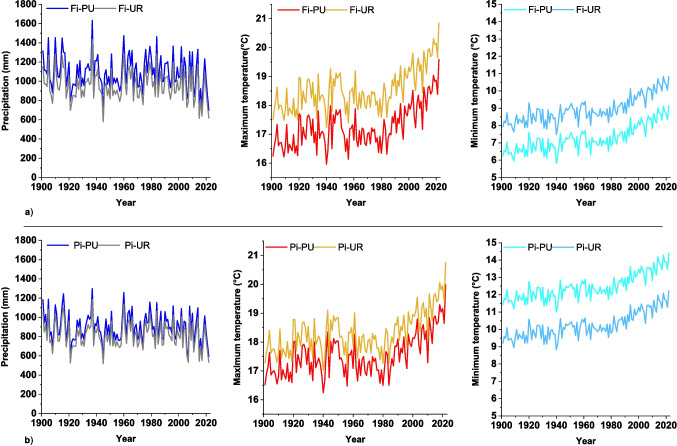
Total precipitation (mm, grey and blue lines), annual maximum temperature (°C, yellow and red lines) and annual minimum temperature (°C, light and dark blue) for **a** periurban “Fi-PU” and urban “Fi-UR” sites in Firenze and **b** periurban “Pi-PU” and urban “Pi-UR” sites in Pisa

The average annual mean maximum temperature increased while total annual precipitation decreased from the 1980 s in four sites (Fig. 1). Before the 1980 s, the annual mean maximum temperature ranged from 15.9 to 17.9 °C in periurban sites and from 17.2 to 19.2 °C in urban sites. Over the last 40 years, the range expanded to 16.3–19.6 °C for periurban sites and 17.5–20.9 °C for urban ones. Moreover, both urban sites experienced higher (> 1.5 °C) maximum temperatures and lower (< 100 mm) total precipitation compared to periurban sites.

### Tree-ring width

**Fig. 2:**
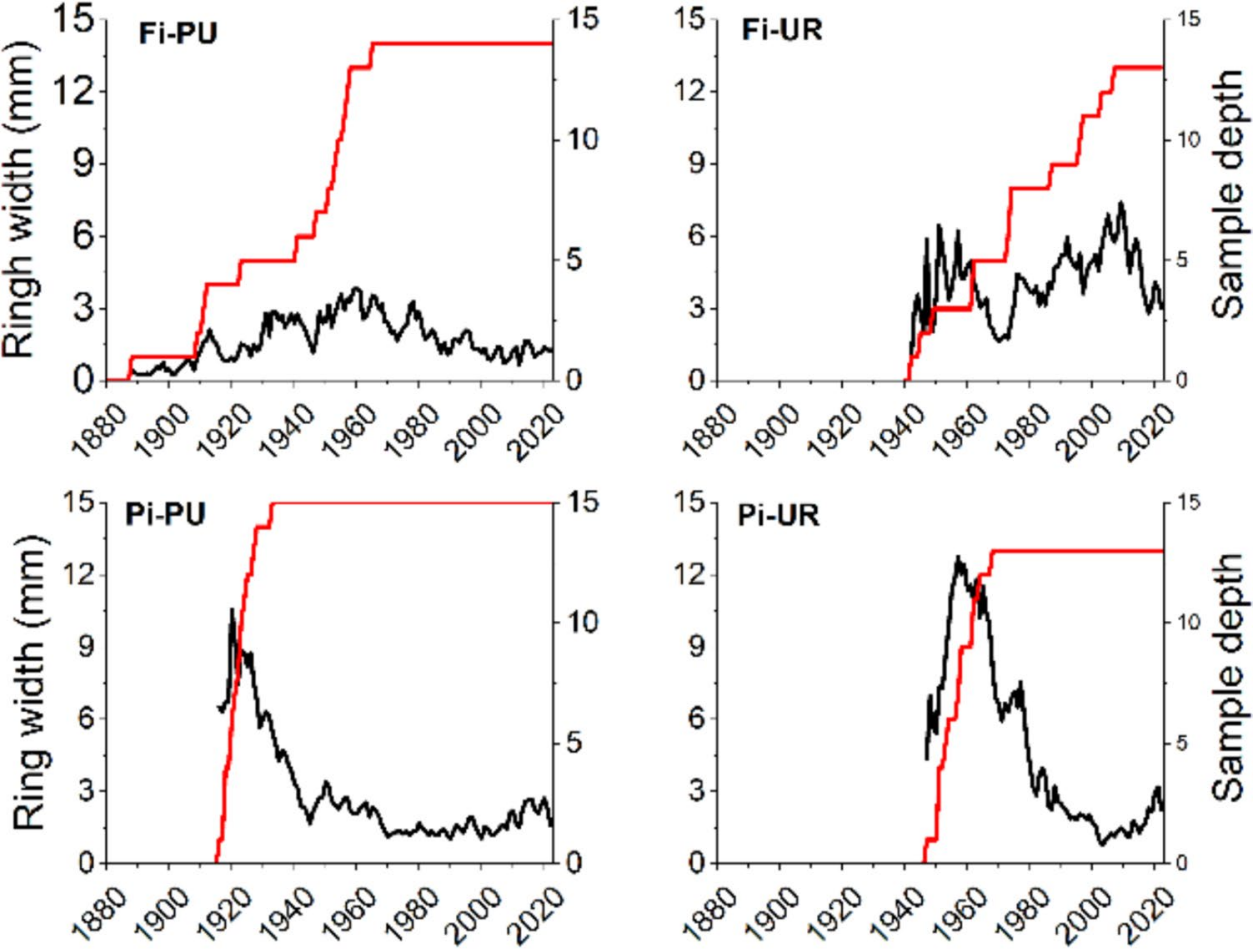
Average ring-width chronologies and sample depth for each site (Firenze periurban “Fi-PU” and urban “Fi-UR” sites; Pisa periurban “Pi-PU” and urban “Pi-UR” sites). The red line refers to sample depth, the black line to ring width

**Fig. 3:**
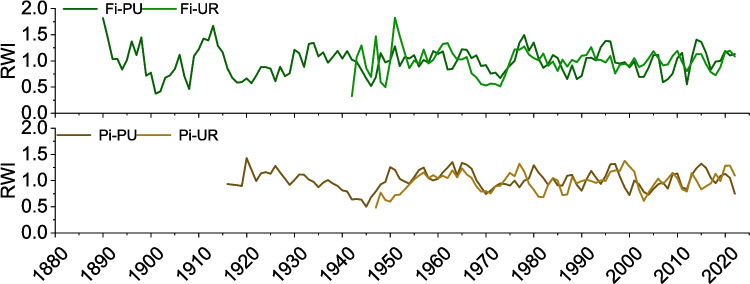
Ring Width Index (RWI) for the two different cities. The top panel shows data from Firenze, and the bottom panel from Pisa

Average ring-width curves and sample depth for the four selected sites are depicted in Fig. 2. Tree-ring chronologies indicated differing ages between periurban and urban sites in both cities (Fig. 2). The initial years of the oldest trees were 1888 at Fi-PU, 1942 at Fi-UR, 1916 at Pi-PU, and 1947 at Pi-UR. Except for Fi-PU, high ring-width variability was observed at the start of each chronology, potentially due to limited sample availability during that period. Furthermore, strong age trends were evident in the two Pisa sites, but not in the Firenze sites. Gleichläufigkeit values were higher in periurban sites compared to urban sites, with mean GLKs of 76% and 77% for Fi-PU and Pi-PU, respectively, and 71% and 72% for Fi-UR and Pi-UR. Negative growth peaks were observed in the 1940 s and 1970 s across all four sites, particularly in the periurban sites, likely due to droughts in those summers. However, RWI (Fig. 3) did not differ significantly between periurban and urban sites in both cities, ranging from 0.44 to 1.71 in Fi-PU, 0.33 to 1.72 in Fi-UR, 0.59 to 1.35 in Pi-PU, and 0.55 to 1.33 in Pi-UR (Table 1).

**Table 1:** Descriptive statistics for the Fi-PU, Fi-UR, Pi-PU, and Pi-UR RWI series

Site	Year	Mean	SD	Median	Min	Max	Range
Fi-PU	135	0.97	0.23	0.96	0.44	1.71	1.27
Fi-UR	81	0.97	0.23	0.99	0.33	1.72	1.38
Pi-PU	107	0.98	0.14	0.99	0.59	1.35	0.75
Pi-UR	76	0.96	0.16	0.98	0.55	1.33	0.77

For each series, the table reports the number of valid observations (year), mean, standard deviation (SD), median, minimum (Min), maximum (Max), and range

**Fig. 4:**
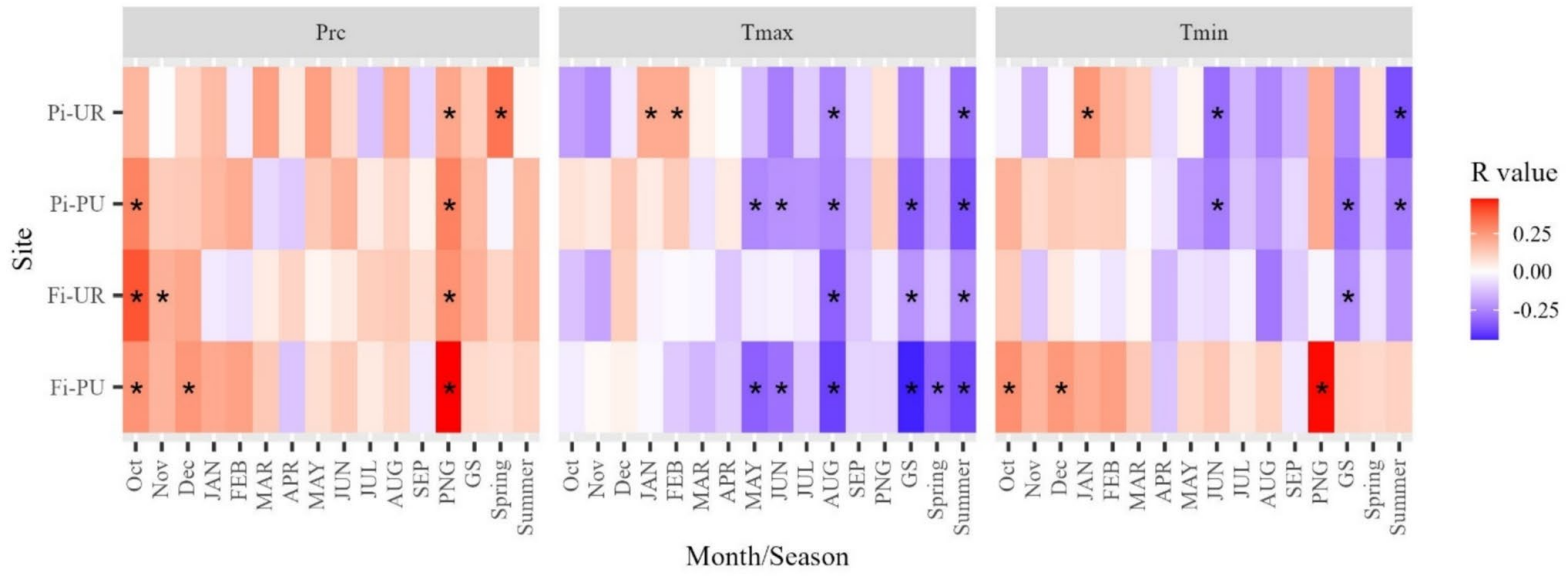
Correlations between RWI and climate data (precipitation, Prc; maximum temperature, Tmax; and minimum temperature, Tmin) for all months from October of the previous year to September of the current year and previous non-growing season, growing season, spring, and summer in periurban “Fi-PU” and urban “Fi-UR” sites in Firenze and periurban “Pi-PU” and urban “Pi-UR” sites in Pisa. Significant correlations are indicated with an asterisk “*”. Cell colour corresponds to the *R*-value scale

Correlation analysis results between RWI and climate data (precipitation, maximum and minimum temperature) are presented in Fig. 4 for all months starting from October of the previous year to September of the current year, as well as for previous non-growing seasons, growing seasons, spring, and summer. RWI exhibited significant positive correlations with precipitation in October of the preceding year in Fi-PU, Fi-UR, and Pi-PU. Furthermore, RWI showed correlations with precipitation in the previous November in Fi-UR, in the preceding December in Fi-PU, throughout the entire prior non-growing season (October–March) across all four sites, and during spring (March–May) in Pi-UR. Regarding maximum temperature (Tmax), negative correlations dominated during mid to late summer months (May to September) across all sites. For minimum temperature (Tmin), significant positive correlations were observed in Fi-PU during October, December, and throughout the entire preceding non-growing season. Fi-UR and Pi-PU exhibited negative correlations between RWI and Tmin during the growing season. Additionally, Pi-PU and Pi-UR showed significant negative correlations between RWI and Tmin in June and throughout the entire summer season (Fig. 4).

No significant correlation was found between ring width and the isotopic ratios of nitrogen, carbon, and oxygen for the corresponding 5-year block of rings (data not shown).

### δ^13^C

**Fig. 5:**
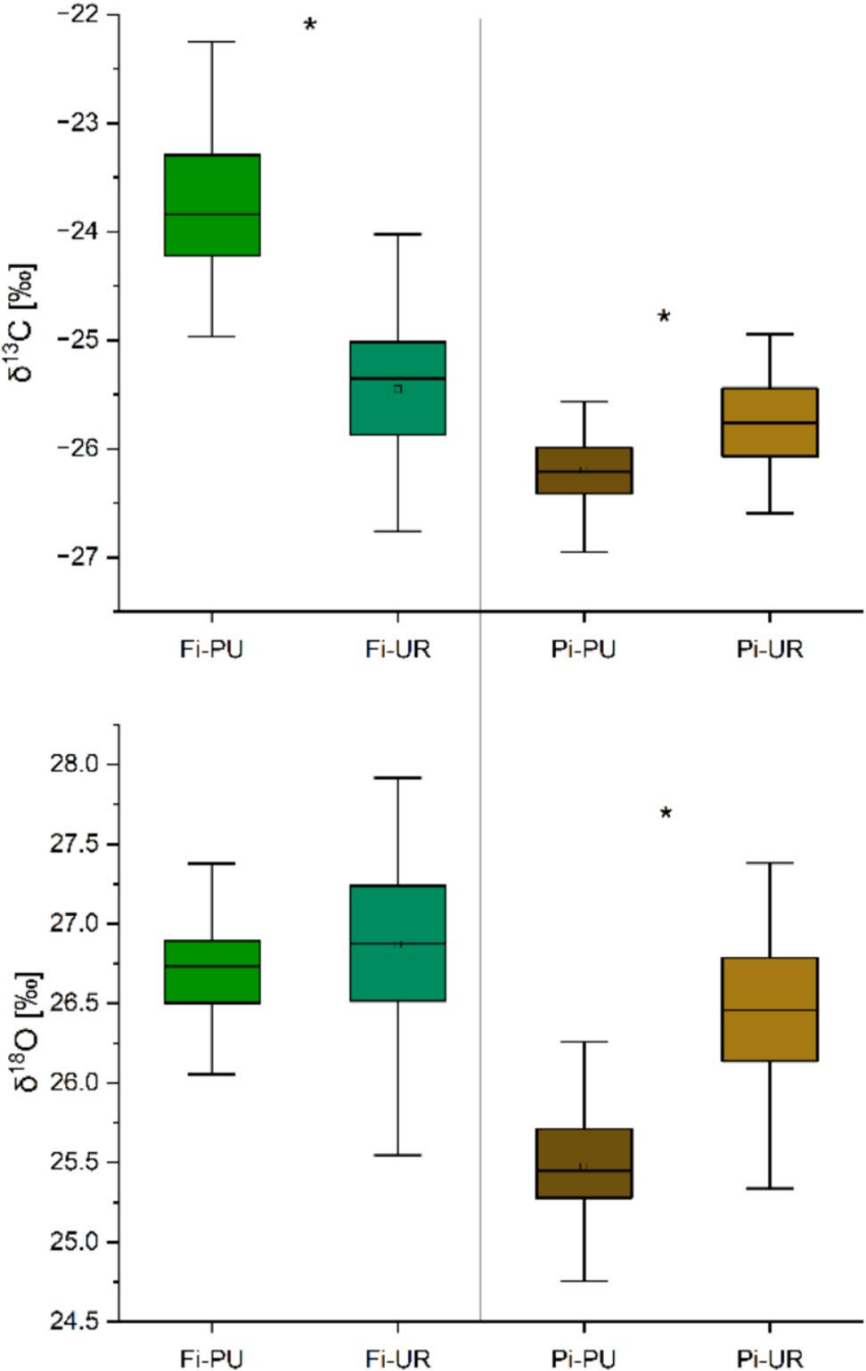
δ^13^C (top) and δ^18^O (bottom) values of urban and periurban sites in Firenze and Pisa (mean values for the period of 1952–2022). Significant differences among sites are marked with asterisks “*”

**Fig. 6:**
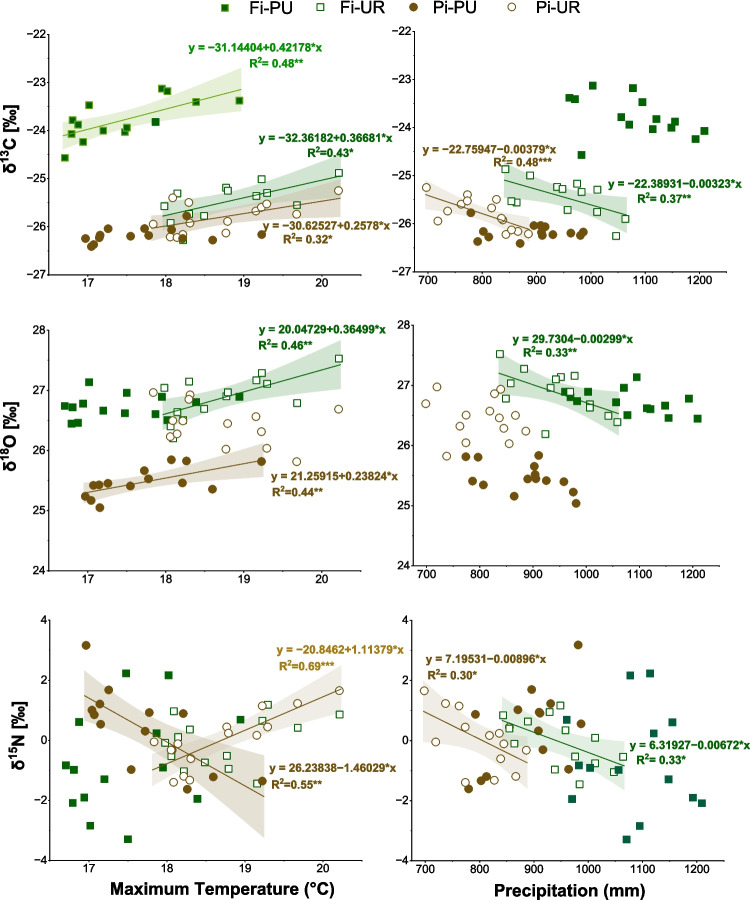
Correlations between δ^13^C, δ^18^O and δ^15^N and the average maximum annual temperature and the sum of cumulative annual precipitation in the corresponding 5-year period in periurban “Fi-PU” (green square) and urban “Fi-UR” (white square) sites in Firenze and periurban “Pi-PU” (brown circle) and urban “Pi-UR” (white circle) sites in Pisa. Significant correlations are indicated by *R*^2^, *p*-values (*p < 0.05; **p < 0.01; ***p < 0.001), trend lines, equations, and confidence intervals. Green equations refer to Firenze (Fi), and brown ones refer to Pisa (Pi)

The mean δ^13^C values for periurban and urban sites were different both in Firenze (− 23.78 ± 0.41‰ in Fi-PU; − 25.46 ± 0.38‰ in Fi-UR), and in Pisa (− 26.17 ± 0.16‰ in Pi-PU, and − 25.80 ± 0.31‰ in Pi-UR) (Fig. 5). A positive correlation between δ^13^C and maximum temperature was observed in Fi-PU, Fi-UR, and Pi-UR (Fig. 6). Additionally, a negative correlation between precipitation and δ^13^C was found for both urban sites but was absent for the periurban sites (Fig. 6).

### δ^18^O

**Fig. 7:**
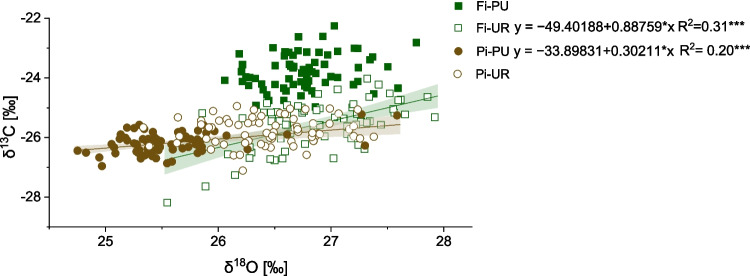
Correlations between δ^13^C and δ^18^O values in tree rings in periurban “Fi-PU” (green square) and urban “Fi-UR” (white square) sites in Firenze and periurban “Pi-PU” (brown circle) and urban “Pi-UR” (white circle) sites in Pisa. Significant correlations are indicated by *R*^2^, *p*-values (*p < 0.05; **p < 0.01; ***p < 0.001), trend lines, equations, and confidence intervals. Green equations refer to Firenze (Fi), and brown ones refer to Pisa (Pi)

The average δ^18^O values in tree rings were lower in Pi-PU (mean ± SD, 25.57 ± 0.30‰) than in Pi-UR (mean ± SD, 26.43 ± 0.33‰) throughout the entire period considered (1953–2022). The values did not differ in Firenze sites (Fig. 5). Positive correlations between δ^18^O and maximum temperature (Fig. 6) were found in Fi-UR and Pi-PU. However, a negative correlation between δ^18^O and precipitation was observed only in Fi-UR (Fig. 6). A moderate and a weak positive correlation were shown between δ^18^O and δ^13^C in Fi-UR and Pi-PU, respectively, suggesting a grouping effect in the latter case (Fig. 7).

### δ^15^N

**Fig. 8:**
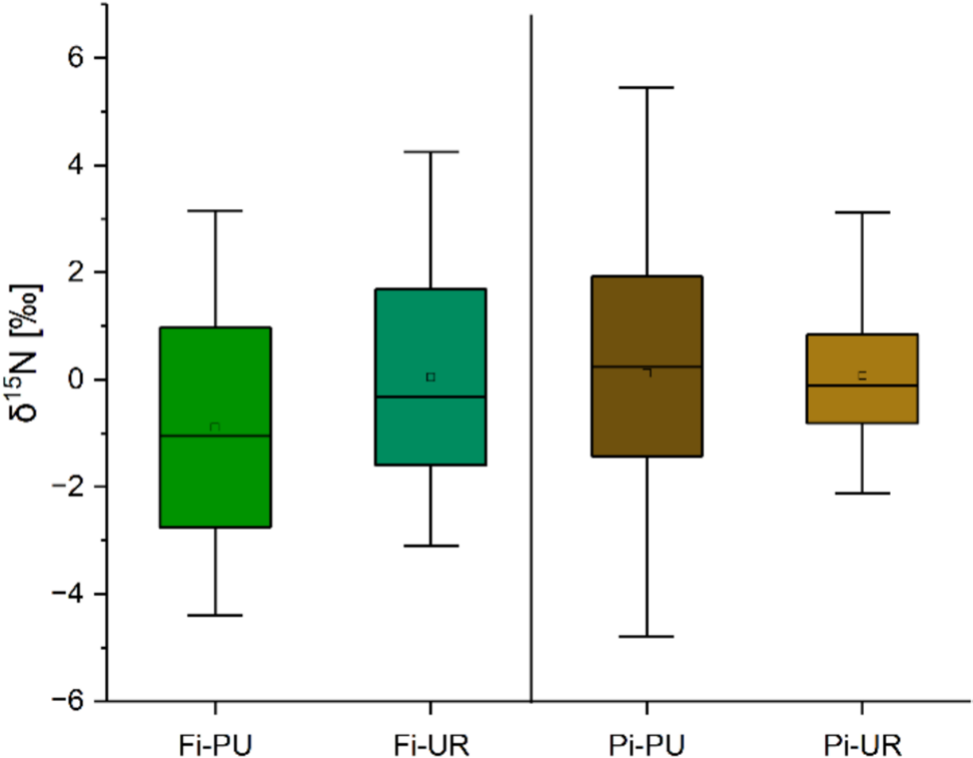
Comparison of δ^15^N values of urban and periurban sites in Firenze and Pisa, based on the mean isotopic values for the period of 1952–2022. Significant results are marked with asterisks “*”

**Fig. 9:**
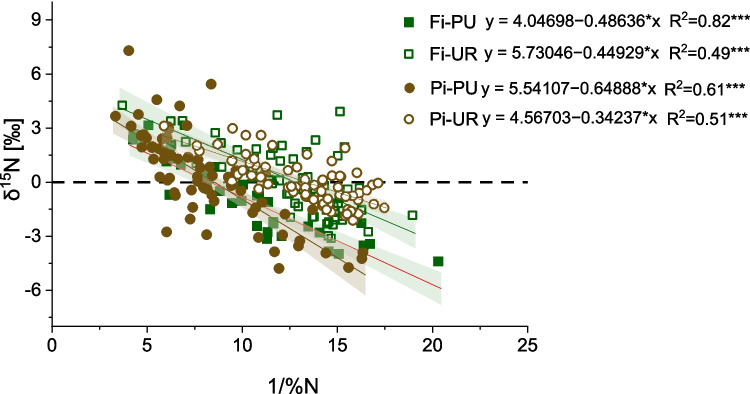
Correlation between the inverse of N (%) concentration (1/%N) and δ^15^N in the tree rings in periurban “Fi-PU” (green square) and urban “Fi-UR” (white square) sites in Firenze and periurban “Pi-PU” (brown circle) and urban “Pi-UR” (white circle) sites in Pisa. Significant correlations are indicated by *R*.^2^, *p*-values (*p < 0.05; **p < 0.01; ***p < 0.001), trend lines, equations, and confidence intervals. Green equations refer to Firenze (Fi), and brown ones refer to Pisa (Pi)

No significant difference in δ^15^N was found among the four sites during the considered period (Fig. 8), although both urban sites showed lower, not significant, N concentrations; 0.11% (± 0.04), 0.08% (± 0.03), 0.13% (± 0.04), and 0.07% (± 0.02) are the mean values of %N in Fi-PU, Fi-UR, Pi-PU, and Pi-UR, respectively. A similar trend of δ^15^N and N-concentration was found in all four sites. Furthermore, a linear relationship was found between δ^15^N and 1/N-concentration, with correlation coefficients of *r* =  − 0.91 in Fi-PU, *r* =  − 0.70 in Fi-UR, *r* =  − 0.78 in Pi-PU, and *r* = 0.71 in Pi-UR (*p* < 0.01, *n* = 14) (Fig. 9). The δ^15^N value of the emissions attributed to NO_*x*_ is represented by the *y*-intercept of the regression line (see “ Sample preparation and stable isotopic measurements” section), i.e., 4.04 (‰) in Fi-PU, 5.73(‰) in Fi-UR, 5.54 (‰) in Pi-PU, and 4.57(‰) in Pi-UR.

Correlations were found between δ^15^N and the average maximum annual temperature and the cumulative annual precipitation (Fig. 6). A negative correlation was found between δ^15^N and the average maximum annual temperature in Pi-PU, while a positive correlation was found for Pi-UR. For both urban sites, the δ^15^N value is negatively correlated with precipitation.

### F^14^C

**Fig. 10:**
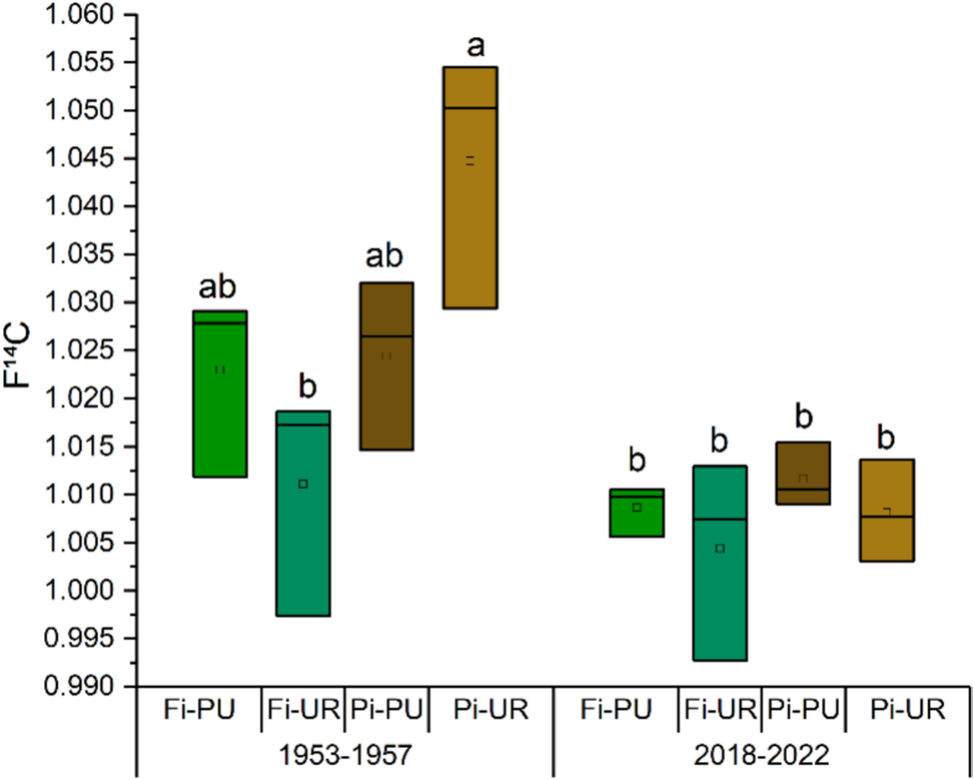
F^14^C (mean ± SE) in the first (1953–1957) and (2018–2022) 5-year blocks in periurban “Fi-PU” and urban “Fi-UR” sites in Firenze and periurban “Pi-PU” and urban “Pi-UR” sites in Pisa. Significant differences in the means resulting from two-way ANOVA are shown with different letters

The results of the F^14^C values are given in Fig. 10. The measured F^14^C values for 1953–1957 are already influenced significantly by ^14^C introduced anthropogenically by nuclear bomb tests, leading to a strong rise in ^14^C over the analysed years (Nydal 1966; Reimer et al. 2004; Pacheco-Solana et al. 2023). The observed variations for the different sites are most likely simply due to variations in the uptake of the bomb signature and are hardly influenced by fossil fuel emissions.

In contrast, the samples analysed for the years 2018–2022 are less influenced by ^14^C introduced by nuclear bomb tests and therefore could be more sensitive to fossil fuel emissions that are depleted in ^14^C. In the last 5-year block, the F^14^C was equal in all four sites and higher than the atmospheric value for the same years (1.000623 ± 0.000168) calculated using data from the Jungfraujoch station (Emmenegger et al. 2023) and considering the summer period (May, June, July and August) of the 5 years from 2018 to 2022.

## Discussion

This study combined dendroecological and isotope analyses to evaluate urban trees’ responses to weather conditions and traffic exposure. Urban sites in Firenze and Pisa were characterized by higher temperatures and lower precipitation than the periurban sites. This difference between urban and periurban areas remains relatively constant from 1901 to the present, particularly pronounced for air temperature. This suggests that it may be related to the complex morphology of the urban environment, defined by buildings and the use of different construction materials, rather than pollution or recent increases in urban atmospheric temperature.

The present study reveals that the RWI did not differ significantly between periurban and urban sites in both cities. Recent studies comparing the growth rates of trees in densely developed urban environments with those in rural forests have produced varied findings. Several studies have documented an earlier onset of phenological phases in urban areas compared to rural regions (Roetzer et al. 2000; Jochner et al. 2013). Additionally, factors such as higher CO_2_ levels (Gregg et al. 2003; George et al. 2009), increased annual nitrogen deposition (Gregg et al. 2003), and the Urban Heat Island effect (Pretzsch et al. 2017) in cities are believed to promote faster tree growth. On the contrary, some recent studies revealed that the urban environment, drought events, and climate change strongly affect and reduce urban tree growth (Brune 2016; Rötzer et al. 2021; Franceschi et al. 2023). However, our results agree with Pretzsch et al. (2017), who did not find a significant difference between urban and rural tree growth, neither before, nor after 1960, under a Mediterranean climate, where negative and positive effects of rural and urban zones seem to be neutralized.

Regarding the climate’s effect on tree growth, the correlation analysis revealed clear seasonal relationships between the RWI and meteorological data. Positive correlations with precipitation, particularly in the preceding autumn and non-growing season, suggested that moisture availability during these periods played a crucial role in supporting tree growth across most sites. This highlights the importance of water supply in the months leading up to and during dormancy for subsequent growth performance. Precipitation plays a key role in regulating cambial activity in the Mediterranean environment (Cherubini et al. 2003). Several studies have found that dry conditions from October to April are the main climatic factors limiting tree growth in this region (Bachtobji et al. 2017; Zhong et al. 2022; Zywiec et al. 2017). Tree-ring growth increases with wet previous autumns and late winters before tree-ring formation, as observed also in *Pinus nigra* by Camarero et al. (2013). This is consistent with the results at all sites, where a significant positive correlation with precipitation during the PNG was found. Conversely, maximum temperature (Tmax) exhibited consistent negative correlations during the warmer mid-to-late summer months, indicating that elevated temperatures during this critical growth period may impose stress and potentially limit radial growth. From a physiological perspective, the delayed positive effect of rainfall on radial growth may reflect the adverse impact of reduced precipitation on carbohydrate reserves (Pallardy 2010). Conversely, elevated spring–summer temperatures in the current year are likely to intensify evapotranspiration (Campelo et al. 2006; Oliveras et al. 2003). In both cases, these climatic constraints can reduce carbohydrate production, thereby reducing tree growth (Liphschitz et al. 1984). Concerning minimum temperatures (Tmin), significant positive correlations during the non-growing season at Fi-PU suggested a beneficial effect of higher minimum temperatures in these months, possibly by extending metabolic activity (Kurz-Besson et al. 2016). However, negative correlations between Tmin and RWI during the growing season and summer in both urban sites and Pi-PU sites imply that higher nighttime temperatures could exacerbate drought stress and negatively impact growth.

Inter-annual δ^13^C values in wood are known to show strong correlations with annual climatic conditions and an increase in response to reduced water availability (Cherubini et al. 2021). This fits with our results in urban trees, as a strong negative relationship between δ^13^C and precipitation was found, along with a positive response to temperature, indicating drought stress. Interannual variations in δ^13^C of urban trees appear more sensitive to high temperatures and low precipitation in both cities than in peri-urban trees. On the contrary, in the latter cases, no clear effect on the carbon isotope ratio was observed due to a non-mutual dependence on temperature and precipitation in Fi-PU. Thus, periurban areas represent a *P. pinea* ecological optimum, as climatic factors do not significantly impact tree health and vitality. However, the higher temperatures and lower precipitation of urban areas represent a likely future climate scenario; the Mediterranean Basin is under critical threat from climate change, and numerous studies highlight its increasing vulnerability to climate change. The Intergovernmental Panel on Climate Change (IPCC) has classified it as a “climate change hot spot,” predicting more frequent and severe climatic events, including rising average and extreme temperatures (Ali and Cramer 2022).

δ^18^O results confirmed the stress response of urban trees, with a positive response to temperature and a negative response to precipitation, at least for Firenze. The oxygen response can be explained by higher evaporative enrichment under dry soil and air conditions (Hirsch et al. 2023). At Fi-UR, the δ^18^O exhibited a climate–response pattern similar to that of δ^13^C, thereby resulting in a positive correlation between the δ^13^C and δ^18^O. δ^13^C variations reflect changes in the ratio of intercellular to ambient CO_2_ concentration (Cᵢ/Cₐ) during the year of ring formation, driven by both stomatal regulation and photosynthetic assimilation rates (Francey and Farquhar 1982; Hubick and Farquhar 1989). δ^18^O values are primarily determined by the isotopic composition of source water and by evaporative enrichment in leaf water, the latter being strongly affected by stomatal conductance and atmospheric humidity during the same period (Gessler et al. 2014). The positive correlation between the two isotopes in Fi-UR indicates stomatal control as the dominant driver of their variability, reflecting periods of increased water stress that correspond to a reduced stomatal closure to discriminate carbon isotope and to an oxygen isotope enrichment. Conversely, low (Pi-PU) or no correlation (Fi-PU and Pi-UR) between isotope ratios suggested that photosynthetic capacity variations or changes in source water isotopic composition played a role in indicating other stress factors than water limitation.The combined isotope information would therefore suggest that the urban trees from Firenze were more drought-stressed than those from Pisa or periurban sites.

The pollution signal (δ^15^N) assessed in this study did not show differences between urban and periurban sites, even though urban sites were selected in the city centre with the expectation of a strong influence of traffic pollution. Interestingly, nitrogen content in the wood was higher at periurban sites, while the δ^15^N pollution signal remained consistent across all sites, resembling values previously observed near motorways (Saurer et al. 2004). This unexpected lack of urban–periurban difference in δ^15^N may be explained by the location of urban sampling sites within parks, which could act as a physical barrier, partially shielding trees from direct traffic emissions and thus reducing the local deposition of NOₓ. Supporting this, Hirsch et al. (2023) reported higher δ^15^N values in trees located near roads and exposed to elevated NOₓ traffic emissions. Similarly, Mifsud et al. (2021) found that *Pinus halepensis* trees nearer to roads exhibited increased δ^15^N compared to those situated further away. These findings, in accordance with other cited studies, highlight that proximity to traffic sources, rather than broad urban versus periurban classification, drives δ^15^N variation. Additionally, soil conditions, processes and nutrient absorption, which were not assessed in this study, may significantly influence nitrogen content in wood, alongside atmospheric nitrogen deposition, which is probably very similar over a large-scale area, at both urban and periurban sites. In particular, the balance between NH_4_ and NO_3_ in the soil, which can be altered by factors like elevated net nitrification resulting from high nitrogen deposition, may impact the δ^15^N values of the nitrogen absorbed by trees (Savard 2010). As a result, there are many isotope fractionation processes involved in the soil as well as within the tree, and therefore several uncertainties are involved in the interpretation of the δ^15^N results. However, in this study, we used δ^15^N as a proxy to understand how much the trees seem to take up NOx. Given that the tree species sampled across urban and periurban sites are the same (*Pinus pinea*), species-specific physiological differences affecting nitrogen uptake and isotope discrimination are expected to be minimal. Therefore, while individual physiological differences can occur, the assumption of a small species-specific effect on δ^15^N values is reasonable in this context, but we cannot completely rule out the importance of NO_x_ pollution as a regulator of tree growth.

The F^14^C results support the results about δ^15^N. Presently (2018–2022), no difference exists between urban and periurban sites in both cities. In addition, atmospheric F^14^C was even lower than in the wood, opposite to what would be expected when the trees take up fossil CO_2_. Even if the atmospheric value calculation estimation is limited by the simplification of the vegetative period we took into account, these findings strongly suggest that urban trees had minimal incorporation of fossil-derived CO_2_, with their F^14^C signatures closely matching or even exceeding calculated atmospheric values.

Thus, the urban climate, characterized by higher temperatures and lower precipitation, may profoundly influence tree physiology more than pollution. This aligns with the findings of Hirsch et al. (2023), who showed that all studied species in urban areas were strongly impacted by drought, as evinced by δ^13^C and δ^18^O signals. The study also noted higher δ^15^N levels in trees situated closer to roads and exposed to higher NOx traffic emissions, though these conditions did not affect tree physiology.

## Conclusion

This study represents one of the first attempts to merge dendroecological and isotope analyses to explore the response of urban trees to high temperatures, low precipitation, and traffic pollution. Focusing on a single species, this study is a first step towards understanding the effects of the urban environment on urban trees. The sensitivity of *P. pinea* to high temperatures and low precipitation, expressed by RWI and isotopes results in urban settings, may hinder its ability to provide ecosystem services and enhance city livability. Understanding these dynamics is essential for predicting urban tree resilience under future climate scenarios. In the face of climate change, urban greening efforts should prioritize species capable of thriving in urban conditions to ensure benefits and promote adaptable, resilient urban planning. In this context, future research should involve different species and plant organs to monitor plants’ eco-physiological responses to urban environments. Furthermore, the study underscores the importance of expansive urban green spaces like parks, located away from main roads, for maintaining air quality comparable to periurban areas. To enhance air quality assessments in our city and parks, additional studies should consider various urban areas such as busy roads with high traffic volumes, as well as industrial and airport zones.

## Data Availability

This is not applicable.
